# Dataset of near-infrared spectral data of illicit-drugs and forensic casework samples analyzed by five portable spectrometers operating in different wavelength ranges

**DOI:** 10.1016/j.dib.2022.108660

**Published:** 2022-10-07

**Authors:** Ruben F. Kranenburg, Yannick Weesepoel, Martin Alewijn, Sharon Sap, Peter W.F. Arisz, Annette van Esch, Peter H.J. Keizers, Arian C. van Asten

**Affiliations:** a Dutch National Police, Forensic Laboratory, Unit Amsterdam, Kabelweg 25, Amsterdam 1014 BA, the Netherlands; b Van't Hoff Institute for Molecular Sciences, University of Amsterdam, Postbus 94157, Amsterdam 1090 GD, the Netherlands; c Wageningen Food Safety Research part of Wageningen University and Research, Akkermaalsbos 2, Wageningen 6708 WB, the Netherlands; d Dutch Customs Laboratory, Kingsfordweg 1, Amsterdam 1043 GN, the Netherlands; e Netherlands Forensic Institute (NFI), Laan van Ypenburg 6, Den Haag 2497 GB, the Netherlands; f National Institute of Public Health and the Environment (RIVM), Antonie van Leeuwenhoeklaan 9, Bilthoven 3721 MA, the Netherlands; g Co van Ledden Hulsebosch Center (CLHC), Amsterdam Center for Forensic Science and Medicine, Postbus 94157, Amsterdam 1090 GD, the Netherlands

**Keywords:** Illicit-drug analysis, Raw spectral data, NIRS, Diffuse reflection, Remission spectroscopy, Forensic science, Powder analysis, Drugs of abuse

## Abstract

The increasing amount of globally seized controlled substances in combination with the more diverse drugs-of-abuse market encompassing many new psychoactive substances (NPS) provides challenges for rapid and reliable on-site presumptive drug testing. Long-established colorimetric spot tests tend to fail due to the unavailability of reliable tests for novel drugs and to false-positive reactions on commonly encountered substances. In addition, handling of samples and chemicals is required. Spectroscopic techniques do not have these disadvantages as spectra are compound-specific and non-invasive tests are possible. Near-infrared (NIR) spectroscopy is a promising technique for on-scene forensic drug detection. Numerous portable devices were introduced in the market in recent years. However, most handheld spectrometers operate in different and relatively confined wavelength ranges compared to the full 780 – 2500 nm NIR wavelength range. In addition, their spectral resolution is limited compared to benchtop instruments. This dataset presents the NIR spectra of 430 forensic samples, including regularly encountered illicit-drugs, NPS, commonly used adulterants, bulking-agents and excipients, and seized casework materials (powders and tablets). Data is available from 5 different NIR spectrometers; including a benchmark high-resolution, full range 350–2500 nm laboratory grade instrument and 4 portable spectrometers operating in the ranges of 1300–2600 nm, 1550–1950 nm, 950–1650 nm and 740–1070 nm. Via this dataset, spectra of illicit-drugs become available to institutes that typically do not have access to controlled substances. This data can be used to develop chemometric detection and classification models for illicit-drugs and provide insight in diagnostic spectral features that need to be recorded for reliable detection models. Additionally, the high-resolution, full range VIS-NIR spectra of the benchmark ASD instrument can be used for *in-silica* predictions of spectra in a certain wavelength range to provide insight in the optimal resolution and wavelength range of a prospective portable device.

**Specifications Table**

**Table:** 

Subject	Analytical Chemistry: Spectroscopy
Specific subject area	Analytical and Forensic Chemistry, on-site presumptive detection or identification of controlled substances by portable spectrometers
Type of data	Tables
How the data were acquired	A total of 430 drugs of abuse-related casework samples, including high purity illicit drugs, were analyzed with five different portable near-infrared (NIR) spectrometers, all operating in a different wavelength range and resolution [1]. Samples were scanned as powders stored in glass vials. NIR spectra were recorded by scanning through the bottom of the glass vials that were placed on top of the spectrometer [2,3]. In addition to the powder samples, a separate set of seized ecstasy tablets [4] was scanned as intact tablets. Tablets were analyzed through the plastic bag in which they were contained.
Data format	Raw (non processed data in *.xlxs format) Raw, replicate averaged
Description of data collection	Seven different sample sets containing a total of up to 430 forensic samples were analyzed over 6 analysis days in 2020-2021. All samples within each set were analyzed sequentially on one of the analysis days for individual spectrometers. Samples originated from either pure reference materials or casework materials that were also analyzed by GC-MS and FTIR analysis at the Amsterdam Police laboratory, as specified in **Table 1**[5], [6], [7]. All samples were stored at ambient temperature in the dark.
Data source location	All spectra originate from samples seized by the Dutch National Police within the greater Amsterdam area, The Netherlands. All seizures were performed in 2020 or 2021. For the case samples (set PAM) only seizures with a powder-like appearance (powders, crystals, grains, chunks) and a light color (white, off-white, cream) were included. [2]
Data accessibility	https://doi.org/10.21942/uva.21252300 DOI:10.21942/uva.21252300
Related research article	R.F. Kranenburg, Y. Weesepoel, M. Alewijn, S. Sap, P.W.F. Arisz, A. van Esch, P.H.J. Keizers, A.C. van Asten, The Importance of Wavelength Selection in On-Scene Identification of Drugs of Abuse with Portable Near-Infrared Spectroscopy, Forensic Chem. (2022) 100437. https://doi.org/10.1016/j.forc.2022.100437. [1]

## Value of the Data

• This data provides insight in the applicability and expected selectivity of NIR spectroscopy for the detection of individual drugs of abuse.
• Diagnostic spectral peaks provide insight in the optimal wavelength range to detect a certain substance and therefore aid spectrometer selection without the need of access to all hardware.
• In addition, the data of the high-resolution, full range 350 – 2500 nm spectrometer provides insight in the fine structure of the NIR absorption bands helping in determining the optimal resolution for a spectrometer.
• Forensic experts, data scientists and spectroscopy manufacturers with limited access to controlled substances can use this data to create and test identification models for illicit drug detection.
• Forensic experts can compare NIR spectra of closely related analogues of drugs (new psychoactive substances) for awareness of possible false-positive or false-negative results.
• Data scientists can use the full range, high resolution NIR data to *in-silico* predict illicit-drug spectra for sensors not included in this study, that operate in a confined wavelength range within the NIR.

## 1 Data Description

File ‘metadata.xlsx’ contains the forensic relevant data of each sample. Individual samples are uniquely coded with both one or more letters and a number (*e.g.* C14, D12, PAM103). The letters depict the set where the samples belong to: C – common drugs; D – designer drugs; N – cocaine-negative substances; PAM – Police Amsterdam powdered casework samples; K – calibration set of binary cocaine mixtures; T – tablets; P – crushed, powdered tablets. The number is a unique number within the set. Details of each set are shown in Table 1. Please note that the numbers are not continuous for all sets as some samples are omitted either in the spectral data or in both the meta-data and spectral data due to mass limits (seizure was too small to take a separate sample for research) or non-matching physical properties. For each sample, the metadata table provides the unique code, component (main active ingredient), form (salt or free base) if available, other information such as identified adulterants or excipients in mixtures, the matrix and the color of the sample material. No quantitative information is available for the presence of adulterants or excipients, although their presence in general ranges between 10 and 50 wt%. It must be noted that the list of adulterants may not be complete as this information originates from GC-MS data only. Non-volatile compounds cannot be detected by this technique (*e.g.* inositol, mannitol, sugar, starch) and will therefore be missed.

**Table 1:** Overview of the sample sets uses for the generation of the NIR dataset.

set	size (#)	description	packaging	origin
C	17	common drugs of abuse	glass vials (clear, borosilicate)	high purity casework samples analyzed by Amsterdam Police
D	38	designer drugs	glass vials (clear, borosilicate)	high purity casework samples analyzed by Amsterdam Police
N	40	non-cocaine set, cocaine-related adulterants and other white colored samples possibly encountered in a forensic setting.	glass vials (clear, borosilicate)	reference material (non-drugs) or high purity casework samples, Amsterdam Police (drugs)
PAM	104	forensic samples with a white or off-white color	glass vials (clear, borosilicate)	various seized casework, 2020, Amsterdam Police
K	88	binary mixtures of cocaine HCl with common adulterants, in 10 wt% steps	glass vials (clear, borosilicate)	prepared by Amsterdam Police
T	72	ecstasy tablets (intact) in LDPE plastic bag, 39x MDMA containing, 32x other drugs, 1x reference MDMA	plastic bag (clear, LDPE)	various seized casework, 2020, Amsterdam Police
P	71	crushed ecstasy tablets (powder), 39x MDMA containing, 32x other drugs	glass vials (clear, borosilicate)	same as set T

All other .xlsx files contain raw spectral data. Files are names in the format: ‘XXX_(Avg_)YYY.xlsx’, where XXX are the letters of the sample set, the presence of Avg_ indicates that in this files contains the average spectra of all replicates, YYY is the name of the spectrometer, being either ASD, NeoSpectra, MicroNIR, NIRONE or SCiO. Due to their small set size, spectra of sets N, C and D are combined in single files (named NCD_(Avg_)YYY.xlsx). Set K data is not available for the NIRone spectrometer. This leads of a total set of 5x5-1 = 24 files containing all raw spectral data of individual scans and another 24 files containing the average spectra per unique sample of this data. In each file data is grouped row-wise per sample with the first row indicating the wavelength in nm and the first column showing the sample code.

## 2 Experimental Design, Materials and Methods

Sample material from sets C, D, PAM, T and the illicit-drug containing samples in set N originated from forensic casework material seized by the police within the greater Amsterdam area in The Netherlands. The identity of the samples was established by the laboratory's validated gas chromatography – mass spectrometry (GC-MS) and Fourier transform infrared (FTIR) identification methods. The non-illicit-drug samples in set N and the adulterants used to produce set K originated from commercially available standards as reported elsewhere [3,8]. Set P originated from the material used in set K by crushing ecstasy tablets to powder. Spectral data was acquired on the five NIR spectrometers operating in different wavelength ranges as specified in Table 2. Scans were recorded by placing the sample (contained in the packaging specified in Table 1) directly on top of the optical head of the sensor [2,3]. Samples were removed and subsequently replaced on the sensor between replicate scans. All vials contained at least a 5 mm thick layer of sample material. Spectra were stored in absorbance values. For the SCiO, no units were provided for the raw data and data was stored as provided by the instrument platform. Background reference measurements were performed according to instructions provided by the manufacturer. For the ASD, MicroNIR and SCiO a dedicated white reference was supplied with the instrument. For the NeoSpectra and NIRone a Spectralon™ reference was used. Raw data was exported in comma separated values using the instrument software and set-wise combined in Microsoft Excel files.

**Table 2:** Overview of the NIR spectrometers used to collect this dataset.

instrument	vendor	wavelength range (nm)	raw data output (datapoints)	reported resolution (nm)
ASD LabSpec 4	Malvern Panalytical, Malvern, UK	350–2500	2151	10
NeoSpectra	Si-Ware, Cairo, Egypt	1300–2600	160	16
NIRone 2.0	Spectral Engines, Steinbach, Germany	1550–1950	201	15 - 21
MicroNIR	Viavi Solutions, Scottsdale, AZ, USA	950–1650	125	12.5
SCiO	Consumer Physics, San Fransisco, CA, USA	740–1070	331	n.a.

## Ethical Approval

This study presents spectroscopic data obtained from casework samples seized by the Dutch National Police. The study was approved by the head of the Forensic Investigation Department of the Amsterdam Police, and Mr. Kranenburg was granted permission to use the anonymized laboratory data for scientific publication as formalized in a collaboration agreement between the University of Amsterdam and the Amsterdam Police. Both the Amsterdam Police and the Science Faculty of the University of Amsterdam hold a license for analytical chemical research on illicit substances issued by Farmatec on behalf of the Ministry of Health, Welfare and Sport of the Netherlands.

## CRediT authorship contribution statement

**Ruben F. Kranenburg:** Resources, Methodology, Investigation, Data curation, Writing – original draft. **Yannick Weesepoel:** Methodology, Investigation, Data curation, Formal analysis, Writing – original draft. **Martin Alewijn:** Formal analysis. **Sharon Sap:** Investigation. **Peter W.F. Arisz:** Writing – review & editing. **Annette van Esch:** Investigation. **Peter H.J. Keizers:** Investigation. **Arian C. van Asten:** Supervision, Writing – review & editing.

## Declaration of Competing Interest

The authors declare that they have no known competing financial interests or personal relationships that could have appeared to influence the work reported in this paper.

## Data Availability

Zip file: Dataset of Near-Infrared Spectral Data of Illicit-Drugs and Forensic Casework Samples Analyzed by Five Portable Spectrometers Operating in Different Wavelength Ranges (Original data) (FigShare).
